# Automatic Determination of Validity of Input Data Used in Ellipsoid Fitting MARG Calibration Algorithms

**DOI:** 10.3390/s130911797

**Published:** 2013-09-05

**Authors:** Alberto Olivares, Gonzalo Ruiz-Garcia, Gonzalo Olivares, Juan Manuel Górriz, Javier Ramirez

**Affiliations:** 1 Department of Signal Theory, Telematics and Communications, CITIC-University of Granada, Calle Periodista Rafael Gómez Montero, 2, E-18071 Granada, Spain; E-Mails: gorriz@ugr.es (J.M.G.); javierrp@ugr.es (J.R.); 2 Department of Computer Architecture and Computer Technology, CITIC-University of Granada, Calle Periodista Rafael Gómez Montero, 2, E-18071 Granada, Spain; E-Mails: gruiz@ugr.es (G.R.-G.); gonzalo@ugr.es (G.O.)

**Keywords:** calibration, accelerometer, magnetometer, MARG, MEMS, automatic, validation, fuzzy, FLS, thresholding

## Abstract

Ellipsoid fitting algorithms are widely used to calibrate Magnetic Angular Rate and Gravity (MARG) sensors. These algorithms are based on the minimization of an error function that optimizes the parameters of a mathematical sensor model that is subsequently applied to calibrate the raw data. The convergence of this kind of algorithms to a correct solution is very sensitive to input data. Input calibration datasets must be properly distributed in space so data can be accurately fitted to the theoretical ellipsoid model. Gathering a well distributed set is not an easy task as it is difficult for the operator carrying out the maneuvers to keep a visual record of all the positions that have already been covered, as well as the remaining ones. It would be then desirable to have a system that gives feedback to the operator when the dataset is ready, or to enable the calibration process in auto-calibrated systems. In this work, we propose two different algorithms that analyze the goodness of the distributions by computing four different indicators. The first approach is based on a thresholding algorithm that uses only one indicator as its input and the second one is based on a Fuzzy Logic System (FLS) that estimates the calibration error for a given calibration set using a weighted combination of two indicators. Very accurate classification between valid and invalid datasets is achieved with average Area Under Curve (AUC) of up to 0.98.

## 1. Introduction

The use of Microelectromechanical (MEMS) Magnetic, Angular Rate and Gravity (MARG) sensors has exponentially increased during recent years, leading to an estimated $3, 500M business in 2013 and a predicted $9, 000M business by 2017 [1]. Many commercial electronic devices such as smartphones, tablets, GPS navigators, watches and videogame peripherals include accelerometers and magnetometers that are used to estimate the orientation of a body in space [2–4], compute trajectories [5–7], analyze motion [8–10], and other innovative measurement applications such as 3D scanning [11].

MEMS MARG sensors need to be calibrated in order to transform measured raw data into meaningful physical units and/or to compensate for a series of undesired effects. Calibration is, thus, a very important step to improve the accuracy of the measurements and, consequently, to increase the quality and efficiency of the system in which the sensors are embedded. A large amount of works regarding accelerometer and magnetometer calibration algorithms has been published over the last decade. Some representative examples can be found in [12–20].

Most calibration algorithms are based on 3-D ellipsoid fitting methods, which minimize a cost function to estimate the value of a set of calibration parameters that define the sensors model [21]. Once the calibration parameters are estimated, they are substituted in the general equation that models the sensor to obtain the calibrated measurement. Every calibration algorithm requires the operator to carry out a set of maneuvers to gather the necessary data for the minimization process. These maneuvers usually require placing the sensor in multiple random positions in the space trying to cover the locus of a sphere, which has a radius equal to the norm of the physical magnitude being used as a reference (Earth's magnetic and gravitational fields or a constant angular speed). The resulting 3D representation of the gathered points is shaped as an ellipsoid since the intrinsic errors in the sensor's output distort the ideal sphere.

Covering all the positions to define a perfectly populated ellipsoid is not a straightforward task as it is complicated to keep a visual record of all the positions that have already been swept. As a consequence, it is usual to gather sets of points that are ill-distributed and lack important parts of the ellipsoid.

**Figure 1.**
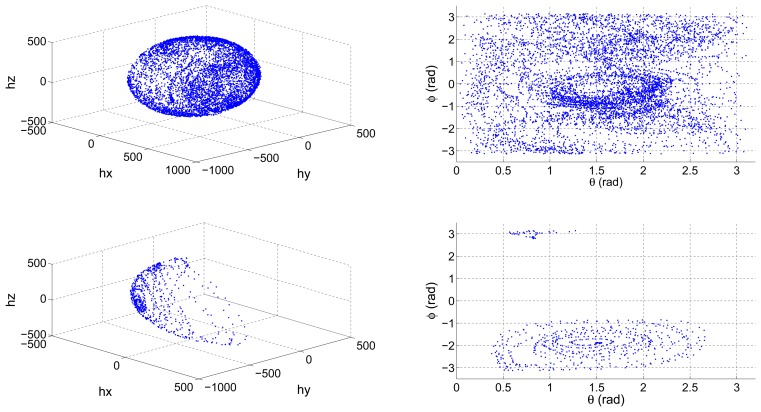
Gathered input magnetometer calibration datasets: well distributed (**top row**), ill-distributed (**bottom row**). 3D representation (**left column**) and projection of spherical coordinates (**right column**).

On the other hand, some navigation systems recalibrate their sensors periodically. If the vehicle in which the navigation system is installed has not covered enough positions relative to a reference frame, then the data that are continuously being gathered can lead to an erroneous calibration. Lotters *et al.* [22] presented an in-use calibration algorithm, which periodically recalibrated an Inertial Measurement Unit (IMU) attached to a human body. It lacked from a mechanism of validation of the distribution of the data being gathered so as the authors acknowledge, if a certain number of positions of the body had not been covered, the resulting calibration error was very high. Figure 1 shows a properly distributed set of magnetometer data, and an incomplete set resulting from an erroneous performance of the calibration maneuvers.

Ill-distributed calibration sets result in a less defined ellipsoid which, in turn, results in a suboptimal set of calibration parameters estimated by the ellipsoid fitting algorithm [22]. It is then very important to use properly distributed calibration sets to ensure the correct estimation of the calibration parameters and an efficient calibration process of MARG sensors. Therefore, it is also desirable to have a system that constantly analyzes the distribution of the data that will be used to recalibrate the parameters and only enable the calibration process when a low error is guaranteed.

Camps *et al.* [23] proposed a solution to help the operator carrying out the calibration maneuvers. They developed a data visualization tool, which depicted in real time the value of the spherical coordinates *ϕ* and *θ* as it is depicted in the right column of Figure 1. This way, the operator has instant feedback on which areas have not yet been covered and can, therefore, place the sensor in the remaining positions.

This is a simple and valid solution if there is a base station to which the sensor sends the calibration data in real time. However, what happens if we want to gather in-field calibration data and we do not have any equipment to act as a base station? What happens if IMU, or the development board containing the sensors, stores the data locally and does not have the ability to transfer data in real time? In these two cases, the feedback visualization tool would be of no help.

A possible solution is to develop an automatic algorithm that is able to determine the validity of the calibration dataset by analyzing the spatial distribution of the data while it is being gathered. To do so, we will need to identify a series of indicators that can link the spatial distribution of input calibration data to the resultant calibration error that would be obtained if such a dataset was provided to the calibration algorithm. The calibration error can be defined in many ways, e.g., the error of the estimated calibration parameters with respect to the optimal ones, or the error of the norm of the calibrated measured physical magnitude with respect to the norm of a known reference. In this work, we opted to use the latter definition as it will be further detailed in the experiments section.

### 1.1. Objectives

The main objectives of this work are listed below:

- Find a series of indicators that contain information about the spatial distribution of the input calibration data of MARG sensors.
- Develop an automatic system that uses one or a combination of various indicators to validate the calibration dataset and ensure a proper calibration process.

### 1.2. Paper Structure

The paper is structured as follows. Section 2 includes the basics of ellipsoid-fitting calibration algorithms as well as models used to describe the output of MARG sensors. Section 3 presents the selection of indicators and the proposal of two algorithms, one using a single input and based on a simple threshold comparison, and another one using a multiple input and based on a fuzzy system. Section 4 includes all the experiments that were carried out to test the proposed approaches and a selection of the most significant results. Section 5 contains a discussion of the obtained results and the validity of the developed system. Finally, conclusions are drawn in Section 6.

## 2. Ellipsoid-Fitting Calibration Algorithms

Ellipsoid fitting algorithms are a recurring solution [24–26] to calibrate MARG sensors. These algorithms are applied to estimate a set of parameters that model the output of the sensor and its intrinsic errors. A simple and commonly used model that maps the raw output of a triaxial MARG sensor to the actual value of the measured physical magnitude (acceleration, magnetic field or angular rate) is as follows,

(1) $$\left(\begin{array}{c} v_{x}\\ v_{y}\\ v_{z} \end{array}\right)=\left(\begin{array}{ccc} k_{x} & 0 & 0\\ 0 & k_{y} & 0\\ 0 & 0 & k_{z} \end{array}\right)\;\left(\begin{array}{c} u_{x}\\ u_{y}\\ u_{z} \end{array}\right)+\left(\begin{array}{c} o_{x}\\ o_{y}\\ o_{z} \end{array}\right)$$

where *v_i_* represents the raw sensor output, *k_i_* is the scale factor (or sensor gain), *u_i_* is the value of the physical magnitude that we wish to estimate and *o_i_* is the offset of axis *i* = {*x*, *y*, *z*}. This equation can be expressed in a more compact way using matrix notation,

(2) v=Ku+o

This model can be complicated by adding other parameters that model undesired effects such as the orthogonality error between sensor axes, *i.e.*, the non-orthogonality between the sensor triplet, or the misalignment error between the axes of the sensor and the reference axes of the boar, and/or the box in which it is mounted.

**Figure 2.**
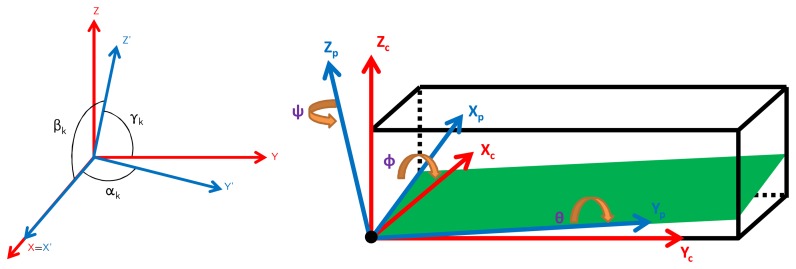
Representation of a non-orthogonal sensor triplet (**left**) and representation of a misaligned circuit board with respect to the IMU's external box (**right**).

The following model proposed by [25] includes the orthogonality and misalignment errors,

(3) v=KTMu+o

where v, K and o, u are again the raw measurements vector, the scale factor matrix, the offset vector and the actual measurement value, respectively. T and M are the non-orthogonality matrix and misalignment matrix and can be expressed as follows,

(4) $${\text{T}}_{k}=\left[\begin{array}{ccc} 1 & 0 & 0\\ \cos\alpha_{\text{k}} & 1 & 0\\ \cos\beta_{\text{k}} & \cos\gamma_{\text{k}} & 1 \end{array}\right]$$

where *α* represents the displacement angle of Y' axis with respect to X axis, *β* the displacement angle of Z' axis with respect to X axis and *γ* the displacement angle of Z' axis with respect to Y axis. In a nutshell, the orthogonalization matrix T_k_ transforms the vector expressed in the orthogonal sensor reference frame *XY Z* into the vector expressed in the non-orthogonal sensor reference frame XYZ'. The misalignment matrix M*_k_* is a rotation matrix, composed of three individual axis rotation matrices, which converts the frame X*_c_*Y*_c_*Z*_c_* in a rotated frame X*_p_*Y*_p_*Z*_p_*. Both effects are depicted in Figure 2.

(5) $$\begin{array}{c} \begin{array}{ccc} {\text{M}}_{\text{K}}=\left[\begin{array}{ccc} 1 & 0 & 0\\ 0 & \cos\phi_{\text{k}} & \sin\phi_{\text{k}}\\ 0 & -\sin\phi_{\text{k}} & \cos\phi_{\text{k}} \end{array}\right] & \left[\begin{array}{ccc} \cos\theta_{\text{k}} & 0 & -\sin\theta_{\text{k}}\\ 0 & 1 & 0\\ \sin\theta_{\text{k}} & 0 & \cos\theta_{\text{k}} \end{array}\right] & \left[\begin{array}{ccc} \cos\psi_{\text{k}} & \sin\psi_{\text{k}} & 0\\ -\sin\psi_{\text{k}} & \cos\psi_{\text{k}} & 0\\ 0 & 0 & 1 \end{array}\right] \end{array}\\ {\text{M}}_{\text{k}}=\left[\begin{array}{ccc} \cos\theta \cos\psi_{\text{k}} & \cos\theta_{\text{k}}\sin\psi_{\text{k}} & -\sin\theta_{\text{k}}\\ \sin\phi_{\text{k}}\sin\theta_{\text{k}}\cos\phi_{\text{k}}-\cos\phi_{\text{k}}\sin\psi_{\text{k}} & \sin\phi_{\text{k}}\sin\theta_{\text{k}}\sin\psi_{\text{k}}+\cos\phi_{\text{k}}\cos\psi_{\text{k}} & \sin\phi_{\text{k}}\cos\theta_{\text{k}}\\ \cos\phi_{\text{k}}\sin\theta_{\text{k}}\cos\psi_{\text{k}}+\sin\phi_{\text{k}}\sin\psi_{\text{k}} & \cos\phi_{\text{k}}\sin\theta_{\text{k}}\sin\psi_{\text{k}}+\sin\phi_{\text{k}}\cos\psi_{\text{k}} & \cos\phi_{\text{k}}\cos\theta_{\text{k}} \end{array}\right] \end{array}$$

where *ϕ*_k_, *θ*_k_ and *ψ*_k_ are the rotation angles around X', Y' and Z' axes respectively.

Now, knowing that the norm of the actual measurement u is constant under stable magnetic conditions (in the case of the magnetometer), quasi-static conditions (in the case of the accelerometer), constant turning rate (in the case of the gyroscope), we obtain,

(6) $$U^{2}=u_{x}^{2}+u_{y}^{2}+u_{z}^{2}$$

where *U* is the norm of the magnetic, angular rate or acceleration reference. Substituting Equation (1) in Equation (6), we get

(7) $$U^{2}={\left(\frac{v_{x}-o_{x}}{k_{x}}\right)}^{2}+{\left(\frac{v_{y}-o_{y}}{k_{y}}\right)}^{2}+{\left(\frac{v_{z}-o_{z}}{k_{z}}\right)}^{2}$$

which happens to be the equation of an ellipsoid with center o + epsilon and semiaxes *k_x_*, *k_y_* and *k_z_*.

The goal of ellipsoid-fitting algorithms is to estimate the set of parameters defined in the sensor model in order to compute the value of the calibrated measurement in Equation (1) or (3). The estimation of the parameters *p* = (*k_x_*, *k_y_*, *k_z_*, *o_x_*, *o_y_*, *o_z_*, …) is usually done by minimizing the mean square error *e*(**p**) between the norm of the estimated calibrated measurement û and the norm of the actual measurement **u**,

(8) $$e(\text{p})=\frac{1}{N}\sum_{n=1}^{N}{\left(\left\|\text{u}\right\|-\left\|\hat{\text{u}}(\text{p})\right\|\right)}^{2}$$

where N is the total number of measurements used for the calibration process (which will we later refer as to the “input calibration dataset”). The norm of the actual measurement ‖**u**‖ is a known magnitude and takes the following value depending on the sensor being calibrated:

- Magnetometer: **u** is equal to the norm of the Earth's magnetic field in the location in which the sensor is being calibrated.
- Accelerometer: **u** is equal to the norm of the Earth's gravitational field in the location in which the sensor is being calibrated. This value is usually normalized to 1 if acceleration is measured in g units.
- Gyroscope: **u** is equal to the value of a constant angular rate to which the sensor is subject, e.g., using a rate table.

The minimization process can be carried out using linear or non-linear optimization algorithms. Another way to estimate the parameters in Equation (7) is to use an enclosing ellipsoid algorithm such as the one in [27].

## 3. Validation Algorithms

Now that we have briefly revised the basic principles of MARG sensor modeling and ellipsoid-fitting calibration algorithms, we can proceed to present the core of our work.

### 3.1. Selection of Indicators

As aforementioned, the main goal of our work is to develop an algorithm that links the spatial distribution of input calibration data to the error obtained after the calibration process. Therefore, we need to analyze the calibration input data to obtain a reliable verdict of the suitability of their spatial distribution. To do so, we need to select a set of indicators that contain relevant information about the spatial distribution of data. We have selected four parameters that we originally thought would provide useful information. The chosen indicators are:

- Indicator A: Sum of the difference of the third quartile (Q3) and the first quartile (Q1) of spherical coordinates *ϕ* and *θ*. A large difference between Q3 and Q1 indicates that data cover a wider region of space and, thus, are not concentrated in a limited region. If the values taken by both *ϕ* and *θ* are homogeneously distributed within [−180°, 180°] and [−90°, 90°] respectively, the value of the indicator will take a high value in the [0°, 540°] range. Therefore, indicator A can be computed as follows,
(9) $${\text{I}}_{A}=(Q3_{\theta }-Q1_{\theta })+(Q3_{\phi }-Q1_{\phi })$$
- Indicator B: Sum of the number of empty bins in the histograms of spherical coordinates *ϕ* and *θ*. Based on the premise of the work carried out by [23], we considered that the analysis of the distribution of the histograms could be a significant indicator. Ideally, an optimal calibration data distribution should contain a large number of well distributed points. Then, the associated *ϕ* and *θ* histograms should contain a reduced number of empty bins. Indicator B can be expressed as,
(10) $$\begin{array}{cc} {\text{I}}_{B}=\#\left\{B_{\theta_{i}}/\#B_{\theta_{i}}=0\right\}+\#\left\{B_{\phi_{i}}/\#B_{\phi_{i}}=0\right\}, & i=1,\ldots ,N \end{array}$$
  where # is the cardinality of a set (number of elements of the set), *B_θ_i__* and *B_ϕ_i__* are the elements of the sets *B_θ_* and *B_ϕ_* composed of the histogram bins of *θ* and *ϕ* respectively, and *N* is the total number of histogram bins.
- Indicator C: Sum of the Euclidean distance between the maximum and minimum values of each of the gathered data components, *i.e.*, the sum of the lengths of axes of the ellipsoid defined by the gathered data. Indicator C is computed using,
(11) $${\text{I}}_{C}=d(\max(g_{x}),\min(g_{x}))+d(\max(g_{y}),\min(g_{y}))+d(\max(g_{z}),\min(g_{z}))$$
  where *g_k_* is the set of values gathered by the sensor in axis *k* = {*x*, *y*, *z*} and defining the set *G*.
- Indicator D: Volume of the convex hull defined by the dataset. The convex hull of a set of G points in the Euclidean space is defined as the smallest convex set that contains G. Therefore, we will be computing the volume contained in the smallest quadric surface defined by the points in the dataset. Indicator D is defined as,
(12) $${\text{I}}_{D}=V(\text{Qhull}(G))$$
  where *G* is the set defined by the gathered points, *Qhull* stands for the Quick Hull algorithm developed by Barber *et al.* [28], and V stands for the volume. The Quick Hull algorithm is available for many programming platforms and languages such as C++, MATLAB, R, GNU Octave, among others. Practical implementation information can be found in [29].

### 3.2. Single-Indicator Thresholding Approach

The first approach that comes to mind is to individually use each one of the selected indicators to check their relation to the calibration error. This approach consists of computing the indicator that is best linked to the calibration error and apply a thresholding algorithm. The thresholding algorithm compares the value of the selected indicator with an empirically predefined threshold and will only validate the calibration dataset if it exceeds it (in the case of indicators A, C and D) or it is under it (indicator B).

(13) $$\begin{array}{cc} \text{IF}\;{\text{I}}_{i}\geq \alpha_{i}; & \text{THEN}\;\varsigma =1 \end{array}$$

where I*_i_* is the dataset indicator for *i* = {*A*, *B*, *C*, *D*}, *α_i_* is the predefined threshold associated to that indicator and *ζ* is the validation flag (*ζ* = 1 if dataset is valid, *ζ* = 0 if not).

A series of experiments are needed to check the strength of the relation of each one of the proposed indicators with the calibration error and their suitability as validators of the goodness of the calibration dataset. In addition, such experiments will also reveal the optimal value of the predefined thresholds. Experiments carried out with this purpose are later explained in Section 4.

### 3.3. Multiple-Indicator Fuzzy Approach

In general, indicators extracted from each set of calibration points are not directly related to the error value, *i.e.*, there is no functional relation between the calibration error and the indicators' values. This means that two different sets of calibration measured points, which may have very similar values for all four geometrical and statistical indicators defined in previous sections, may not have associated error values close to each other. Moreover, it can occur that two calibration sets deriving in similar calibration errors have significantly different indicator values. Despite this non-functional relation between the value of the indicators and the calibration error, it is intuitive and reasonable to think that there exists a fuzzy relation between them. For example, as it will be later demonstrated, a dataset having a large value for the volume comprised by the convex hull and a large distance between components would lead to a low calibration error. The fuzzy relationship between these two indicators (convex hull and distance between components) could be stated as follows,

(14) $$\text{IF}\;{\text{I}}_{c}\;\text{is HIGH AND}\;{\text{I}}_{D}\;\text{is HIGH THEN calibration error IS LOW}.$$

consequently,

(15) $$\text{IF}\;{\text{I}}_{c}\;\text{is LOW AND}\;{\text{I}}_{D}\;\text{is LOW THEN calibration error IS HIGH}.$$

Rules like Equations (14) and (15) can be defined combining all different indicators and defining a complete set of rules. So, if we consider a set of M indicators as the input vector, the calibration error as the scalar output and K sets of calibration points, it is possible to propose a multiple-input single-output function estimation problem from a given set of K input-output pairs.

More precisely, for the calibration set *k*, *k* = 1, …, *K*, let us write each input-output pair as (*x⃗^k^*; *z^k^*), where ${\vec{x}}^{k}=(x_{1}^{k},x_{2}^{k},\ldots ,x_{M}^{k})$ is the input vector comprised by the *M* values of the indicators and *z^k^* is the calibration error. In this work we opted to use a zero-order Takagi-Sugeno-Kang (TSK) Fuzzy Logic System (FLS), defined by a complete set of rules [30] as,

(16) $$\text{IF}\;x_{1}\;\text{is}\;X_{1}^{i_{1}}\;\text{AND}\;x_{2}\;\text{is}\;X_{2}^{i_{2}}\ldots \;\text{AND}\;x_{M}\;\text{is}\;X_{M}^{i_{M}}\;\text{THEN}\;z=R_{i_{1}i_{2}\ldots i_{M}}$$

where $X_{m}^{i_{m}}\in \left\{X_{m}^{1},X_{m}^{2},\ldots ,X_{m}^{n_{m}}\right\}$ are the *n_m_* fuzzy sets of input *m*, and $R_{i_{1}i_{2}\ldots i_{M}}$ is the *i*_1_*i*_2_…*i_M_* scalar consequent of the rule. Choosing the product as the t-norm operator, and denoting $\mu_{X_{m}^{i_{m}}}$ as the membership function of input *m* to the set $X_{m}^{i_{m}}$, the FLS output can be expressed as follows:

(17) $$\tilde{F}({\vec{x}}^{k})=\frac{\sum_{i_{1}=1}^{n_{1}}\sum_{i_{2}=1}^{n_{2}}\ldots \sum_{i_{M}=1}^{n_{M}}\left(R_{i_{1}i_{2}\ldots i_{M}}\prod_{m=1}^{M}\mu_{X_{m}^{i_{m}}}\left({\vec{x}}_{m}^{k}\right)\right)}{\sum_{i_{1}=1}^{n_{1}}\sum_{i_{2}=1}^{n_{2}}\ldots \sum_{i_{M}=1}^{n_{M}}\left(\prod_{m=1}^{M}\mu_{X_{m}^{i_{m}}}\left({\vec{x}}_{m}^{k}\right)\right)}$$

Defining such a fuzzy system would require the following: choosing the number of inputs (M); choosing the number of Membership Functions (MFs) as well as their type and parameters; and selecting the scalar consequents for each rule. Although it is possible to perform all this design manually choosing the value of each parameter, it would be very time expensive, suboptimal and strongly influenced by the designer's subjective criterion. Thus, to avoid these issues, an automatic approach has been selected in this work in order to create an adaptive and nearly optimum FLS.

First of all, we have decided to use triangular MFs comprising a complete partition in the input space [30]. This is a typical choice when designing fuzzy systems [30-32] as it has two main advantages: on the one hand, it avoids the existence of input points that do not activate any rule and cause the FLS to be ill-defined by making the denominator in Equation (17) equal to zero; on the other hand, such a partition makes this denominator always equal to one, which results in a much computationally simpler FLS.

The remaining design decisions are those related to the MFs parameters, *i.e.*, the location of the centers, and the scalar consequents of each rule. As the system will be adaptive, the MFs' centers will change their position, so it is only necessary to specify their initial location. All MFs will shape an equidistant and complete triangular partition. This way, the first and last MFs' centers will coincide with the ends of the input intervals and they will not be selectable parameters as their position is fixed. Finally, the matrix of rule consequents will be initialized to all zeros, and it will change its values in each iteration to reduce the training error, *ε*_train_, defined in the following way,

(18) $$\varepsilon_{\text{train}}=\varepsilon_{\text{calib}}-\tilde{F}$$

where *∈*_calib_ is the actual calibration error that we wish to estimate, which is known during the training process, and *F̃* is the output of the FLS computed using Equation (17).

In general terms, the adaptive strategy adopted in this work is the same as in [32,33]. The consequents matrix *R* is adapted in each iteration following a reward/penalty policy, which changes the values of the consequents of those rules that have a higher responsibility of the present error; whereas the MFs' centers vary according to a gradient descent algorithm, modified by a saturation function.

More precisely, the employed error function is as follows,

(19) $$J(R,C)=\overset{K}{\underset{k=1}{\text{∑}}}{\left(F({\vec{x}}^{k})-\tilde{F}({\vec{x}}^{k};R,C)\right)}^{2}$$

which shows the dependency of *J* on the matrix *R* and the MFs' centers, noted $c_{m}^{i_{m}}$ to indicate the center of the *i_m_* triangular MF of input *m*, which form *C*.

If we consider the quantity *F* (*x⃗^k^*)− *F̃* (*x⃗^k^*; *R*, *C*), it must be noted that, if positive, the output of the FLS should have been greater, whereas if negative, it should have been lower. Therefore, this quantity indicates the magnitude and direction (increase or decrease) that should be applied to each of the consequents of the rules that are responsible for the present value. The rules are not all rewarded/penalized in the same way; but the more active a rule becomes, the greater correction it will receive.

Considering that,

(20) $$\alpha_{i_{1}i_{2}\ldots i_{m}}(\vec{x})=\prod_{m=1}^{M}\mu_{X_{m}^{i_{m}}}({\vec{x}}_{m})$$

is defined as the firing strength or activation degree of a rule, each rule consequent will be modified by the quantity given by,

(21) $$\Delta R_{i_{1}i_{2}\ldots i_{M}}=C\cdot \alpha_{i_{1}i_{2}\ldots i_{M}}(\vec{x})\left(F({\vec{x}}^{k})-\tilde{F}({\vec{x}}^{k};R,C)\right)$$

where *C* is a design parameter [33] that is used in FLS to compensate for the different scales of the approximated system input control signal *u*(*k*) and the output function *y*(*k*) that is used to compute the error. It is usually expressed as,

(22) $$C=\left|\frac{u_{\max}-u_{\min}}{y_{\max}-y_{\min}}\right|$$

However, in this work, the approximated signal is the same as the signal used to compute the error, so we used C = 1.

In the case of the MFs' centers, a similar correction strategy is adopted, this time based on a modified gradient descent algorithm, *i.e.*,

(23) $$\Delta c_{m}^{i_{m}}=-\eta_{m}^{i_{m}}\frac{\frac{\partial J(R,C)}{\partial c_{m}^{i_{m}}}}{\left|\frac{\partial J(R,C)}{\partial c_{m}^{i_{m}}}\right|+l_{m}^{i_{m}}}$$

where $l_{m}^{i_{m}}$ is a learning parameter used to increase the speed of convergence, and,

(24) $$\eta_{m}^{i_{m}}=\{\begin{array}{c} \begin{array}{cc} \frac{c_{m}^{i_{m}}-c_{m}^{i_{m-1}}}{2}, & \text{if}\;\frac{\partial J(R,C)}{\partial c_{m}^{i_{m}}}\geq 0; \end{array}\\ \begin{array}{cc} \frac{c_{m}^{i_{m+1}}-c_{m}^{i_{m}}}{2}, & \text{if}\;\frac{\partial J(R,C)}{\partial c_{m}^{i_{m}}}<0; \end{array} \end{array}$$

This algorithm prevents the centers from changing their order, which in turn prevents the loss of their linguistic meaning. This updating method uses a saturation function based on the gradient magnitude, so the centers can move, at most half the distance from their immediate neighbors.

Finally, it is important to highlight three important issues: first, because of the cumbersomeness of the maneuvers to gather each set of calibration points, the total number of input-output pairs available is not very high (around 50). Such a limited number of points would lead to a very poor fuzzy training. Thus, to avoid this from happening, these 50 points have been used up to 10 several times to provide the fuzzy system with at least 500 hundred points to learn the fuzzy relation. Despite re-usage of these points will not add new information, an artificially larger dataset provides the algorithm with more iterations to improve its convergence.

The second issue to be highlighted is that this training phase would likely need to be computed offline (depending on the power of the processor embedded in the MIMU), presumably in a computer. However, the evaluation function found after the training process can be easily included in the embedded firmware and executed online as it is not computationally expensive.

The third issue to highlight is that although it would have been possible to use optimal consequents adaptation (as in [33]), or even a more complex FLS, it would have not improved the general performance of the validator. Proposing a more complex and capable FLS would only result in a system achieving a lower training error at a higher computational cost, which would neither be able to eliminate unpredictable errors in the output as the relation between markers and calibration error would still be not functional.

## 4. Experiments

**Figure 3.**
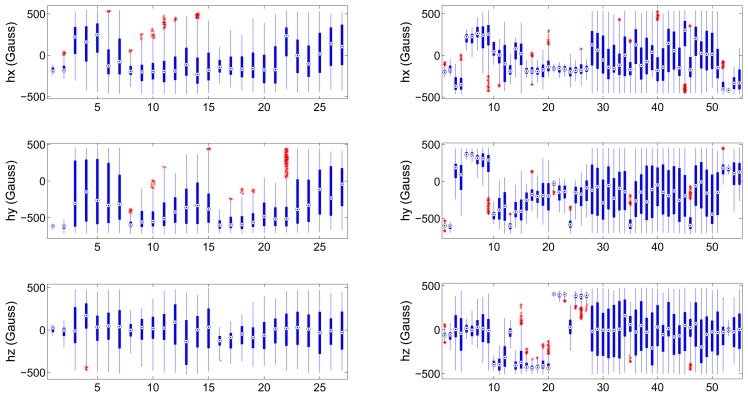
Boxplot of gathered magnetic field components (*h_x_*, *h_y_* and *h_z_*): dataset 1 (**left column**), dataset 2 (**right column**). X axis displays the distribution number.

We have carried out two sets of experiments, one for each of the proposed approaches. We will start by describing the datasets gathered to train and test the validators. We have gathered two groups of calibration distributions; the first one is composed of 27 data distributions and we will refer to it as “dataset 1”; the second group is composed of 55 distributions and we will name it “dataset 2”. Both datasets contain ill-distributed and well distributed input calibration data. Figure 3 depicts the boxplot of each one of the sensing axis (*h_i_*, *i* = {*x*, *y*, *z*}) of a magnetometer for both datasets. This figure reflects the value of the median (circled point), distance between third and first quartiles (length of the thick blue line) and the outliers (red points). It can be seen at a glance the different statistical nature of the gathered distributions.

Once the data to be used are ready, we apply the following pre-processing steps for each one of the calibration distributions:

(i) Compute all four indicators (A, B, C and D).
(i) Execute the ellipsoid-fitting calibration algorithm presented in [23] to estimate the calibration parameters, *i.e.*, scale factors, biases and orthogonality matrix (see [23] for more details).
(i) Apply the computed calibration parameters to calibrate the input calibration data.
(i) Compute the norm of the calibrated data and calculate the error with respect to the value of the norm of Earth's magnetic field in our location (0.432 Gauss [34]). We will refer to this error as the “calibration error”.
(i) Build a vector containing the calibration errors for each one of the distributions of both datasets.
(i) Select a maximum error tolerance (*∈_max_*) from which the associated input calibration distribution will be considered as ill-distributed.
(i) Build a validation binary marker associated to both datasets, which is set to 1 if the distribution is valid and to 0 if not.

**Figure 4.**
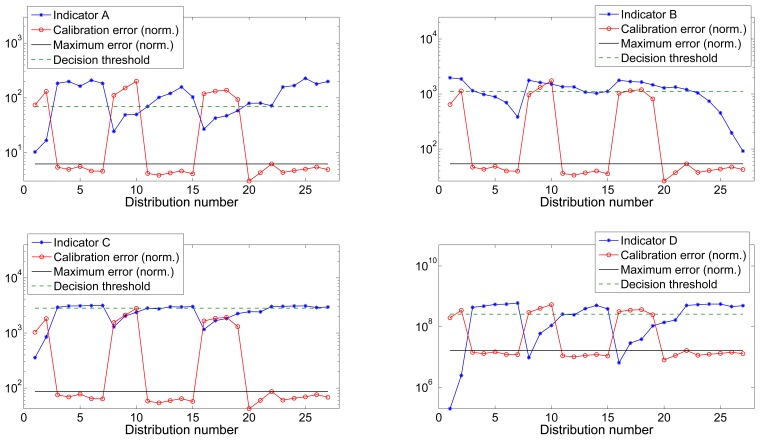
Dataset 1: Indicators *vs.* normalized calibration error.

**Figure 5.**
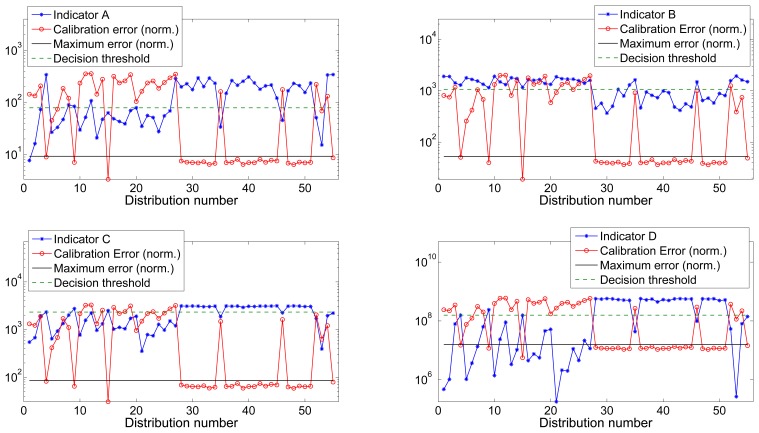
Dataset 2: Indicators *vs.* normalized calibration error.

Figures 4 and 5 show the value of all four indicators *versus* the calibration error for datasets 1 and 2 respectively. Calibration error has been normalized to the maximum value of the indicator to improve the visibility. The maximum calibrated error is the threshold indicating the maximum tolerated error that was used during the training process and to label the distributions as valid or invalid.

Now, once all the auxiliary parameters are ready, the experiments carried out to test the single-indicator thresholding validator are very straightforward. As we explained in Section 3, we compare the value of each one of the indicators with a threshold. Imagine that indicator C has a value of 500 empty bins for distribution *n*. So, if we consider that any distribution having less than, let's say, 1000 bins, will be valid as it will result in a value lower than the maximum tolerated error, then, distribution *n* is marked as valid. Proceeding this way for the *N* distributions of the dataset, we will also build an estimated validation binary marker that is subsequently compared with the actual validation marker. This comparison is carried out by computing the Receiver Operating Characteristic (ROC) curve [35] and the area under the curve (AUC), which are the standard indicators of the performance of binary classifiers.

The value of the predefined threshold has to be set empirically and, in order to find the value resulting in the optimal ROC, we have carried out a grid search. This can be considered as the training process and it has the following steps:

(i) Build a vector containing the initial threshold, the step and the final threshold values for each one of the four indicators.
(i) Apply an iterative process in which every value of the threshold is applied to the indicator in order to build and estimated validation marker and compute the ROC values.
(i) Find the threshold values resulting in the best ROC values *i.e.*, those that maximize the value of the AUC.

The selection of the values of the threshold vectors depend on the characteristics of the employed sensor. In our case, we employed the triaxial magnetometer included in a WAGYROMAG IMU [36]. The measured data are digitalized using a 10 bit ADC, so the range of the measurements is [0, 1023]. The threshold values used for the experiment are listed below. Notice that these values will differ depending on the hardware characteristics of the device being employed.

- Indicator A ((*Q*3*_θ_− Q*1*_θ_*) + (*Q*3*_ϕ_* − *Q*1*_ϕ_*)): as explained in Section 3, the values of *ϕ* and *θ* are distributed within [−180°, 180°] and [−90°, 90°] respectively. Therefore, the possible values of the indicator, and analogously the values of the threshold, are bounded in [0°, 540°].
- Indicator B (Total number of empty bins in *ϕ* and *θ* histograms): since *ϕ* and *θ* can take up to 1,024 values each, the total number of empty bins will be in the [0, 2047] range. Therefore, this will also be the vector of possible threshold values.
- Indicator C (Sum of the Euclidean distance between the maximum and minimum gathered components): Ideally, the radius of the sphere will be in the [0, 512] range. Therefore, the sum of the three ellipsoid axes will be in [0, 3072] range and so will be the threshold vector.
- Indicator D (Volume of the convex hull): given that the volume of a reference well distributed and highly populated raw calibration set is around 5 × 10^8^, while the volume of a poorly distributed set is in the order of 10^7^, then the threshold vector will be set to [1 × 10^6^, 6 × 10^8^].

The selection of a grid-search parameter optimization strategy over any other error minimization algorithm is well justified in this case since the number of possible values the parameters can take is known and limited. Moreover, the grid-search ensures finding the optimal parameters and presents no problems of convergence to local minima as the Gauss-Newton and Levenberg-Mardquart algorithms.

The maximum tolerated calibration error *∈_max_* was chosen by visually inspecting the error vector of both datasets (as depicted in Figures 4 and 5) and choosing the largest between the group of low errors considerable as acceptable. We chose *∈_max_*_1_ = 0.0273 Gauss and *∈_max_*_2_ = 0.0262 Gauss for datasets 1 and 2 respectively. Table 1 shows the optimal threshold values obtained for each one of the indicators for both datasets as well as the associated ROC values.

After the training process, we need to check the validity of the computed parameters using dataset 1 by applying them over dataset 2 and viceversa. Table 2 shows the results derived from this process.

The second part of the experiments is conducted to test the performance of the Multiple-indicator Fuzzy Validator.

We trained the FLS with all the possible input combinations of two indicators and then tested it over the dataset that was not used for the training. The output of the FLS is an estimation of the calibration error values that would be obtained for each one of the input calibration distributions. The estimated calibration error also needs to be compared with a predefined error tolerance, which should ideally be equal to *∈_max_*. Expecting some deviation of the estimated error with respect to the actual error, we also built a vector of error tolerances and carried out a grid-search to find the optimal value obtaining the best ROC parameters.

**Figure 6.**
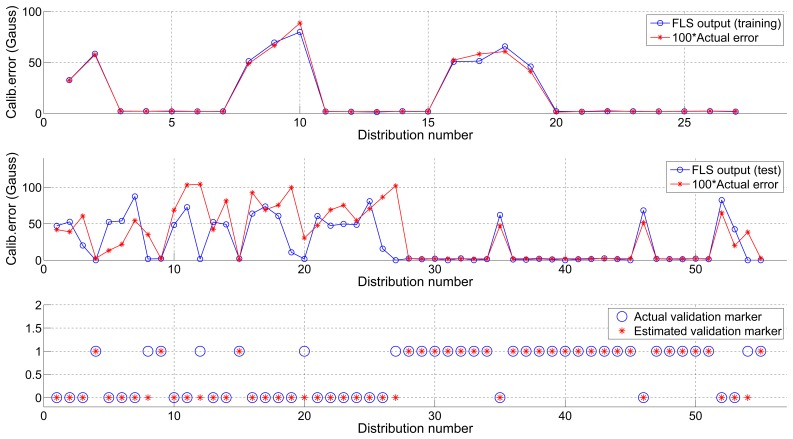
FLS training using dataset 1 (**top**), test using dataset 2 (**center**) and final validation (**bottom**).

Figure 6 shows the outcome of the training process using dataset 1 and its application to dataset 2, as well as the estimated actual binary validation markers. Table 3 reflects the ROC parameters for each one of the combinations together with the optimal error threshold which, as can be seen at a glance, is very close to the actual value (*∈_max_*).

## 5. Results Discussion

When analyzing the results obtained by the Single-indicator Thresholding Validator, we see that for dataset 1 it was possible to find a threshold for all indicators, which ensured perfect classification, *i.e.*, the estimated validation marker was equal to the actual marker. On the other hand, for dataset 2, which included twice the number of distributions, the percentage of correct decisions slightly decreases. However it is still very close to the optimal situation. Regarding the performance of each indicator we observe that:

- Indicator A: It shows the poorest results of all indicators. The sum of the difference between Q3 and Q1 does not consider the possible outliers (extreme values, see Figure 3) which also may contain information about the existence of less populated areas that contribute to cover the complete locus of the sphere and reduce the calibration error. Then, if a distribution contains one or more regions with high density of points and other less populated but still covered areas, the indicator will penalize it although it will likely result in a low calibration error.
- Indicator B: Counting the number of empty bins in the histograms offers an acceptable precision but it can be an ambiguous indicator. Having, for example, two distributions containing a similar number of points, the number of empty bins can be very similar or even identical. However, if one of the distributions covers the complete surface of the ellipsoid with a low density of points, while the other one covers only a limited region of the surface with a high density of points, the calibration error will be very different. The less populated distribution will achieve a lower error since it gives better information to the fitting algorithm about the shape of the ellipsoid.
  This indicator is only trustable when we set a very strict threshold, i.e., when the number of permitted empty bins is very low (∼10% out of the total). By setting a very strict threshold, we can guarantee that the calibration process will yield good results. However, this restriction eliminates valid distributions that have a much lower number of points but offer good performance. In other words, a restrictive threshold causes no false positives (ill-distributed calibration sets being validated) but it may generate a large number of false negatives (well-distributed calibration sets being discarded). If, on the other hand, we set a less restrictive threshold, the number of false negatives will decrease but the number of false positives will increase (which is a less desirable situation).
- Indicator C: The sum of the Euclidean distance between the maximum and minimum values gathered in each axis of the sensor has shown to be the second best indicator among the tested ones. This indicator shows if axis antipodal areas are covered. Then, if it has a high value, it indicates that we have at least covered the three ellipses around the three axes. However, this indicator does not provide further information about the number and density of points in other areas located outside the ellipsoid main axes. The fitting calibration algorithm may have trouble converging to optimal parameters if only points in the axes are provided. In an extreme case in which we only have points contained in the three ellipses around the axes, the indicator will present a high value and tag the distribution as valid while it would actually result in the estimation of erroneous calibration parameters. However, since this is a very extreme and rare case, we can conclude that indicator C is a good indicator of the validity of the input calibration set.
- Indicator D: The volume contained by the smallest surface wrapping the data points (the convex hull) has revealed to be the best of the tested indicators as it contains trustful information about the proper distribution of the calibration sets. It achieved perfect classification for both datasets in the training process (*i.e.*, it was possible to find a threshold value that perfectly distinguished between valid and invalid distributions) and an almost perfect classification in the test process, which was only partially distorted by a reduced number of false negatives (which are less critical than false positives). The computation of the volume solves the situation explained in the previous point in which data points are only contained in the ellipses around the axes. If this is the case, the approximated convex hull will present sharper and more abrupt edges and, hence, the volume will be lower and the set will be penalized.

**Figure 7.**
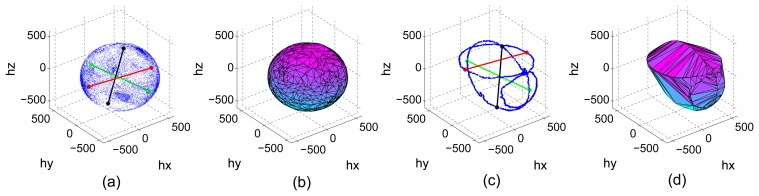
Well distributed dataset: Euclidean distances (**a**), Convex hull (**b**). Ill-distributed dataset: Euclidean distances (**c**), Convex hull (**d**).

Figure 7 shows a well distributed and highly populated set and a set in which the points are only contained in ellipses around the axes triplet. Distances between maximum and minimum components as well as the estimated convex hull are also plotted to see the aforementioned problem. The value of the indicators is *C*_well_ = 3113, *C*_ill_ = 2954, *D*_well_ = 4.9196 × 10^8^ and D_iill_ = 3.9166 × 10^8^. Notice how the difference between *D*_well_ and *D*_ill_ is more accentuated as the difference between *C*_well_ and *C*_ill_, hence, the better discrimination ability of indicator D.

The Multiple-indicator fuzzy approach was developed to test the performance of a decision system using combinations of weighted indicators as input. The FLS showed its power to obtain an estimation of the calibration error using only the indicators as the input during the training phase. The success of the testing phase varied depending on the employed combination of indicators.

The combination of indicators A and B achieved the poorest performance with an average AUC of 0.8963 ± 0.0105 (Table 2). On the other hand, as it was expected, the joint use of indicators C and D obtained an almost perfect decision rate with an average AUC of 0.9861 ± 0.0197 (Table 2) showing the best performance of all tested approaches. Indicators C and D have been proved to contain more relevant information about the goodness of the spatial distribution of the input calibration datasets.

The FLS approach is also easier to tune, as the output is an estimate of the calibration error, so the threshold can be directly set according to the desired degree of calibration error tolerance. On the other hand, it is harder to regulate (manually set) the value of the indicators' threshold when using the Single-indicator approach since the relationship between the value of the indicator and the calibration error is not functional and therefore, not evident.

## 6. Conclusions and Future Work

In this work, we have presented the problems derived from the use of ill-distributed datasets as the input of ellipsoid fitting algorithms used to calibrate MARG sensors. We have presented two different systems that monitor and assess the goodness of the spatial distribution of data being gathered during the calibration maneuvers and, therefore, validate them only when a low calibration error is ensured.

Both approaches are based on the computation and analysis of four distribution indicators, namely the sum of the difference of the third quartile and the first quartile of spherical coordinates *ϕ* and *θ* (indicator A); the sum of the number of empty bins in the histograms of spherical coordinates *ϕ* and *θ* (indicator B); the sum of the Euclidean distance between the maximum and minimum values of each of the gathered data components (indicator C) and the volume of the convex hull defined by the dataset (indicator D).

The first approach, referred to as the “Single-indicator Thresholding Approach” is based on comparing the value of a single indicator with a predefined threshold from which it is considered that the error will be low. We carried out a grid-search training process that revealed the optimal value of the threshold of each indicator. For indicator D, we found a threshold value that ensured perfect classification, *i.e.*, the estimated validation marker was identical to the actual marker. After finding the optimal threshold values, we tested them in a dataset different from the one used for the training. Again, marker D obtained the best performance with an average ROC of 1.0000 ± 0.0000 in the first dataset and 0.8889 ± 0.1571 in the second dataset.

The second approach was designed to use weighted combinations of two indicators as the input. We designed a TSK Fuzzy Logic System with 10 membership functions that was used to infer the relationship between the indicators and the calibration error.

Results showed that the estimation of the error was very accurate and an average AUC of 0.9861 ± 0.0197 was obtained using parameters C and D as the input.

Results have been obtained for a triaxial magnetometer since the data gathering process is faster than for the accelerometer. Accelerometers need to be placed in quasi-static positions to avoid the linear acceleration from distorting the gravity, which is used as the reference. This fact considerably increases the duration of the calibration maneuvers to obtain the same number of points as with the magnetometer. Results are generalizable since when using ellipsoid fitting algorithms the only difference comes from the physical magnitude used as a reference (norm of Earth's magnetic field, norm of Earth's gravitational field or a constant known angular rate).

It has been shown that the calibration error of ellipsoid fitting algorithms can be predicted by analyzing the spatial distribution of the input datasets. The system we propose could be used to improve the performance of automatic in-use calibration algorithms that periodically recalibrate MARG sensors, as well as to ease the calibration maneuvers carried out by in-field operators. In addition, in case that the IMU being calibrated had local processing capabilities, our algorithm could be included in the firmware and send an alert to the operator (through an LED, playing a sound or with any other kind of actuator) whenever the calibration dataset is well distributed, to inform the operator that no further maneuvers are required.

Future work will be oriented to implement an algorithm to detect and reject outliers in data distributions caused by magnetic distortions to improve the performance of our system when applied to magnetometers. Measurements of magnetometers can be disrupted by external magnetic fields other than the Earth's. If the MIMU is used in a very unstable magnetic environment, the magnetometer's output will be distorted and the indicators may get erroneous values, which may fool the detection system. We will also evaluate other alternatives to the FLS such as neural networks and multifactor regression to reduce the computational load of the training process.

## Figures and Tables

**Table 1.** Results of the training process of the Single-indicator Thresholding Validator.

**Training set**	**Indicator**	**Max. Error tol. (*∈_max_*)**.	**Optimal threshold**	**Sensitivity**	**Specifity**	**AUC**	**Average AUC**
1	A	0.0273	59.0000	1.0000	1.0000	1.0000	0.9648 ± 0.0497
2	A	0.0262	109.0000	0.9231	0.9310	0.9297	
1	B	0.0273	1.3530E + 3	1.0000	1.0000	1.0000	0.9947 ± 0.0075
2	B	0.0262	1.3040E + 3	0.9615	1.0000	0.9894	
1	C	0.0273	2.3810E + 3	1.0000	1.0000	1.0000	0.9987 ± 0.0019
2	C	0.0262	2.2440E + 3	0.9615	1.0000	0.9973	
1	D	0.0273	1.1030 + E8	1.0000	1.0000	1.0000	1.0000 ± 0.0000
2	D	0.0262	9.8000 + E7	1.0000	1.0000	1.0000	

**Table 2.** Results of the test procedure of the Single-indicator Thresholding Validator.

**Training Set**	**Indicator**	**Optimal threshold**	**Sensitivity**	**Specifity**	**Average ROC**
1	A	59.0000	1.0000	0.7241	0.8620 ± 0.1951
2	A	109.0000	0.6667	1.0000	0.8333 ± 0.2357
1	B	1.3530E + 03	0.9615	0.8966	0.9290 ± 0.0459
2	B	1.3040E + 03	0.8333	1.000	0.9166 ± 0.1179
1	C	2.3810E + 03	0.9231	1.0000	0.9616 ± 0.0544
2	C	1.2440E + 03	1.0000	0.7778	0.8889 ± 0.1571
1	D	1.1030E + 08	1.0000	1.0000	1.0000 ± 0.0000
2	D	9.8000E + 07	1.0000	0.7778	0.8889 ± 0.1571

**Table 3.** Results of the test procedure of the Multiple-indicator Fuzzy Validator. Datasets 1 and 2.

**Training Set**	**Indicators**	**Max. Error Tol. (*∈_max_*)**.	**Optimal Thr.**	**Sensitivity**	**Specifity**	**AUC**	**Average AUC**
1	A&B	0.0262	0.0250	1.0000	0.8276	0.9038	0.8963 ± 0.0105
2	A&B	0.0273	0.0220	0.7778	1.0000	0.8889		
1	A&C	0.0262	0.0230	0.9615	0.8966	0.8893	0.9168 ± 0.0390
2	A&C	0.0273	0.0230	0.8889	1.0000	0.9444		
1	A&D	0.0262	0.0310	0.9615	0.9310	0.9198	0.9460 ± 0.0371
2	A&D	0.0273	0.0250	0.9444	1.0000	0.9722		
1	B&C	0.0262	0.0360	0.9615	0.8621	0.8548	0.9274 ± 0.1027
2	B&C	0.0273	0.0490	1.0000	1.0000	1.0000		
1	B&D	0.0262	0.0260	0.9615	0.7931	0.7858	0.8790 ± 0.1318
2	B&D	0.0273	0.0240	0.9444	1.0000	0.9722		
1	C&D	0.0262	0.0250	1.0000	1.0000	1.0000	0.9861 ± 0.0197
2	C&D	0.0273	0.0220	0.9444	1.0000	0.9722		
